# Modeling of Curvilinear Steel Rod Structures Based on Minimal Surfaces

**DOI:** 10.3390/ma14226826

**Published:** 2021-11-12

**Authors:** Jolanta Dzwierzynska, Igor Labuda

**Affiliations:** Department of Architectural Design and Engineering Graphics, Faculty of Civil and Environmental Engineering and Architecture, Rzeszow University of Technology, Al. Powstancow Warszawy 12, 35-959 Rzeszow, Poland; labuda@prz.edu.pl

**Keywords:** engineering materials, engineering structures, optimization, genetic algorithms, parametric design, shaping structures, curvilinear steel rod structure, modeling structures, Enneper surface, Grasshopper

## Abstract

The article deals with shaping effective curvilinear steel rod roof structures using genetic algorithms by implementing them for the analysis of various case studies in order to find new and efficient structures with positive characteristics. The structures considered in this article are created on the basis of the Enneper surface and minimal surfaces stretched on four arcs. On the Enneper surface, a single layer grid is used, while on the other surfaces, two-layer ones. The Enneper form structure with four supports and the division into an even number of parts along the perimeter of the covered place proved to be the most efficient, and the research showed that small modifications of the initial base surface in order to adapt the structure to the roof function did not significantly affect its effectiveness. However, the analysis and comparison of single and double-shell rod structures based on minimal surfaces stretched on four arcs have shown that a single-shell structure is much more effective than a double one. The paper considers the theoretical aspects of shaping effective structures, taking their masses as the optimization criterion. The optimization helped to choose the best solutions due to structures’ shapes and topologies. However, the obtained, optimized results can find practical applications after conducting physical tests.

## 1. Introduction

The paper deals with the use of steel as the structural material for modeling curvilinear rod structures. The systematics of steel rod structures is quite complex as they can be differentiated due to their function, shape, kind of material used, as well as prefabrication methods. However, due to their overall shape, that is, the shape of the surface they form, the following types of steel rod structures can be distinguished: flat, single-curved as well as double-curved. On the other hand, in terms of their grid systems, the steel rod structures can be divided into single-layer and multi-layer structures. Geometric grids are characterized by a large topological diversity. However, single-layer coverings usually occur in a curved form, whereas multi-layer coverings are more often applied as flat ones [1]. Modeling/shaping of any steel rod structure can be defined as the continuous optimization of its form and shape due to assumed criteria and constraints. From the architectural point of view, during shaping, the main emphasis is put on functionality and aesthetics, whereas from a structural aspect, shaping can be treated as determination of the system that meets the assumed criteria for strength, which are specified by standards [2,3,4,5]. However, architectural and structural shaping criteria are interdependent [6].

Moreover, as far as curvilinear steel rod roof structures are concerned, they are subjected to the specific geometric shaping criteria for this kind of structure. These criteria relate mainly to geometric parameters of the structure’s module, its height, span, and topology [7].

The stage of shaping of any steel rod structure, as one of the earlier phases of designing, is not only a creative phase, but it also significantly influences the final properties of the shaped structure. In the case of shaping of steel rod structures, one of the known methodologies can be used: minimizing the energy used to produce structural material, minimizing material consumption or analyzing material reuse [8]. Moreover, in the case of shaping steel rod structures, the location of rods in the structure can be determined using both the numerical and geometric methods. In the first method, in order to establish structure nodes’ position, the coordinates of the point grid and directional vectors are used. However, in the second method, the polyhedral base solids can be used, which divide and fill a given space, or divisions of the so-called base surfaces can be applied [9]. Due to this fact, both the shape of the base surface and the kind of its division in order to establish structure’s nodes are important. Historical development and a broad overview and description of various kinds of spatial divisions and topologies of grid structures are presented in [10], where the first surfaces to be divided in order to obtain curvilinear steel rod structures were spherical and cylindrical surfaces [11].

This article is a continuation of the author’s considerations on the shaping of effective steel rod structures, whose geometries were generated by division of the base surfaces using parametric design tools, which were presented in previous publications [9,12,13,14,15]. There, as the base surfaces, the surfaces composed of straight lines have been used, that is, ruled surfaces such as cylindroid or hyperbolic paraboloid, which are advisable due to their easy discretization [9,12,13,14,15].

The ruled surfaces were divided in order to receive meshes. Next, these meshes helped to establish structural models being combinations of beams and shells. The optimal structures in terms of their weights were obtained by genetic optimization methods. Namely, the multicriteria optimization method of shaping canopy roofs of hyperbolic paraboloid and cylindroid shapes using genetic algorithms is presented in [9,15], whereas a single criteria optimization method with the application of genetic algorithms is presented in [12,13,14].

Nowadays, there is a growing interest in the application of evolutionary optimization in structural engineering. Therefore, much research explores the possibility of using genetic algorithms in order to optimize the designed structures of various materials [16,17,18]. In the case of steel structures, they are mostly used for topology optimization to generate innovative lightweight rod structures with interesting and efficient rods configurations that are difficult to obtain in a conventional way [19]. Topological optimization of trusses, steel frames, and grid structures is described in many publications. Truss optimization with the application of a genetic algorithm method is presented in Reference [20]. The procedure and software in order to optimize size, topology and shape of plane trusses using genetic algorithms, as well as finite element analysis to evaluate fitness function, is shown in [21]. Steel truss optimization using hybrid genetic algorithms and finite element analysis FEA is also described in [22]. An efficient algorithm for optimization of the layout of trusses is proposed in Reference [23]. Multi-objective optimization of spatial trusses based on node movement is shown in [24].

On the other hand, the structural topology optimization of shell grid structures is given in References [25,26]. Many recent publications as well as research projects in the field of structural engineering deal with the problem of reducing the impact of loads on the structures by modifying and optimizing their topologies or shapes [27,28]. The finite element method (FEM) is a computational tool for performing such engineering analysis and optimizations. It encompasses the application of mesh generation by dividing a complex problem into small ones, as well as using software that is coded with an FEM algorithm. It allows one to model, analyze and design structures of various materials, e.g., the steel structures, among others [29,30,31,32,33].

The finite element analysis software used in the current research is Autodesk’s structural design software, Robot Structural Analysis Professional [34]. However, the geometry of the structures is generated and optimized by means of the tools for parametric design, that is, Rhinoceros 3D/Grasshopper (Robert McNeel and Associates) [35].

As the base surfaces for the curvilinear steel rod structures’ creation, not only can the surfaces with advantageous features in terms of the possibility of subdivision be selected, such as the ruled surfaces mentioned previously, but also the surfaces with shapes for convenient distribution of stresses under loads, such as minimal surfaces.

According to mathematical definition, minimal surfaces are the surfaces that locally minimize their areas, which is equivalent to having zero mean curvature [36]. They are surfaces with the smallest possible area among all surfaces stretched on the given lines. Minimal surfaces are used mostly to construct light roof structures. Nowadays, due to the beneficial characteristics of minimal surfaces, there is increasing interest in the study of their properties, and there is a tendency to their wider use in order to create innovative architectural objects. Therefore, they can also constitute the base surfaces for steel rod structures’ creation. As the minimal base surface, already known surfaces can be used, such as an Enneper surface or new kinds of free form surfaces. The Enneper surface is mostly investigated as a form for tensioned fabric structures [37]. A lot of works analyze geometric properties of Enneper surfaces, but little is known of the application of these surfaces in the building industry. It is also difficult to find research works considering the Enneper surface as inspiration for shaping steel rod structures, although attempts to shape steel rod structures using the Enneper surface were undertaken by the author in [38]. Modeling the form of the minimal surface can be a big challenge, and it depends, among other things, on the available design tools. Computer Aided Design tools (CAD) used on a large scale in designing cannot be used to create an optimal minimal surface shape, which can constitute the basis for creating any steel rod structure [39]. However, the development of digital modeling tools, especially the tools which enable simulations describing the geometry and behavior of membrane structures, allows the creation of minimal surfaces [40,41].

In this context, the aim of the present study is optimal shaping of curvilinear steel rod structures, assuming minimal surfaces as the base surfaces for structural nodes’ locations. The effective structures are obtained through the appropriate generation and approximation of their geometries and the use of an appropriate rod system.

Namely, in this article, base surfaces are considered both the known minimal surface, such as the Enneper one, and novel free form minimal surfaces determined by four arches. This research is partly an extension of the author’s previous research on shaping curvilinear steel rod structures for which the Enneper surface is considered as a starting base surface, which was presented in [38]. Namely, the effectiveness of the structures based on the Enneper surface was tested assuming three various support variants and three different variants of rod grids. The most effective variant was selected. Current research consists of optimizing the grid topology and modifying the shape of the structure received as the best solution due to research presented in [38]. Its goal is increasing its efficiency, as well as to better adapt it to the intended function. Another type of structure analyzed in the paper is the curvilinear steel rod structures based on the free form minimal surfaces stretched on four arches. In this case, both single shell and multi-shell surfaces are considered, which are the base surfaces for the generation of 3D trusses. The comparison of single shell and double shell structures is performed. The aim of the research is to check how the geometry of the minimal base surface influences the load transmission by structures and how to obtain efficient steel rod structures by modification of their geometries and topologies. However, the originality of the research consists of showing how to take advantage of a combination of parametric tools and finite element analysis software during shaping structures in order to obtain interesting and effective structures.

## 2. Materials and Shaping Methods

In general, shaping of structures began with the creation of parametric, geometric models of minimal base surfaces.

In order to generate minimal base surfaces, the parametric shaping tools in the Rhinoceros 3D environment (Robert McNeel and Associates) [35] were applied. These tools enable the generation of various free forms based on Non-Uniform Rational B-Splines (NURBS). Especially, Grasshopper was used, which is an algorithmic modeling tool representing a graphical programming language integrated with Rhinoceros 3D. Grasshopper enables the creation of block scrips determining parametric, geometric models of the considered structures. Next, these models can be modified, optimized by simulations, analyzed and after any modifications presented visually in the Rhinoceros’s viewport.

In this research, the calculation models of structures were defined based on the geometric, discretized models formed by means of Grasshopper. The grid lines were regarded as beams, whereas grid vertices became the structural nodes. Assumed boundary conditions regarding a supporting system and load conditions, as well as material and joint properties, were specified too. Finally, the structures were analyzed and optimized by means of Robot Structural Analysis Professional software due to dead and environmental loads [34]; however, as the optimization criterion, the structures’ masses were used.

The structures modeled and analyzed in the research were the structures of the network composed of straight steel members creating a single layer or a double layer 3D grid. As the structural material for all considered structures, Structural Steel of EU Grade S235 was used. However, as the covering, glass panels of density equal to 2400 kg/m³ and thickness of 6 mm were applied or polycarbonate plastic sheets for roofing of density equal to 0.02 kg/m^3^ and panel thickness equal to 10 mm. In the case of single layer lattices, rigid joints have been applied, whereas in the case of a 3D double-layer grid, pinned joints.

In this research, two various approaches to shaping steel rod structures have been applied dependently on the kind of base surface used, as specified below. The shaping approach in the case of structures formed on the basis of the Enneper surface:

- Generation of a parametric, geometric model of the Enneper surface with 4 supporting points over a circular place by means of Grasshopper,
- Adjusting the parameters describing the surface to the design assumptions,
- Division of the surface along the radius and along the places’ perimeter into an even number of parts,
- Creation of the single layer grid model and their modification in order to better adapt them to the function of the roof covering structures,
- Determination of 3 structural models with defined boundary conditions,
- FEA of the structural models and their optimization by means of Robot Structural Analysis Professional software.

Shaping approach in the case of structures formed on the basis of the free form minimal surface:

- Generation of a parametric, geometric model of the free form minimal surface stretched over four arcs and covering a rectangular place by means of Grasshopper,
- Adjusting the parameters describing the surface to the design assumptions,
- Optimization of the surface’s shape and area as well as its adaptation to the function of the roof covering by means of Grasshopper,
- Division of the surface and determination of the two-layer grid topologies,
- Determination of single-shell and multi-shell structural models with defined boundary conditions,
- FEA of the structural models and their optimization by means of Robot Structural Analysis Professional software.

## 3. Results

### 3.1. Shaping of Steel Rod Structures Based on the Enneper Surface

The Enneper surface is one of the representatives of the minimal surfaces group. According to the algebraic geometry definition, it is a self-intersecting surface, which can take various interesting shapes depending on the choice of parameters that define it. The subject of the present analysis is a curved steel rod structure constituting a roof covering over a round place, whose nodes are included in the Enneper surface.

The shaping process starts from a proper definition of the Enneper surface’s model. Due to the round shape of the covered place, the parameters describing the surface are adjusted in such a way as to receive a regular, non-intersecting shape of the Enneper surface whose horizontal projection is a circle.

Next, for the need of the creation of Grasshopper’s algorithms describing a single layer 3D grid model, the Enneper surface is described by two parameters (u, v). The parameter u is measured along the place’s radius, while the parameter v is measured along the places’s perimeter. These parameters determine the mesh composed on the surface and in this way the shape and topology of the grid formed on the given surface.

Due to the fact that this research is a continuation of the previous research, some observations have been taken into account when making the initial assumptions for shaping [38].

**Figure 1:**
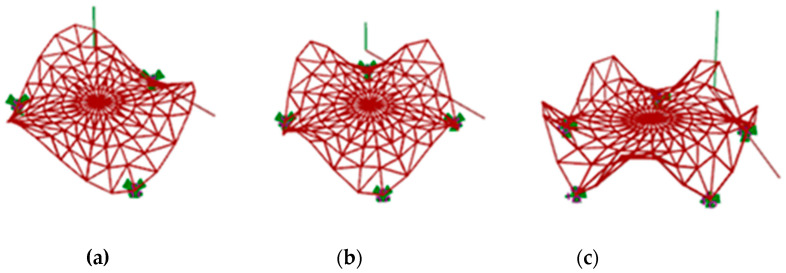
Various models of curvilinear rod structures based on the Enneper surface: (**a**) with three supports; (**b**) with four supports; (**c**) with five supports.

Previously, various steel rod structures with different topologies based on the Enneper surfaces have been analyzed, covering a round place and characterized by three, four or five supports [38]. The research has proved that the single layer structure with four supports and topology presented in Figure 1 is the most efficient: characterized by both the smallest mass and displacement. Assuming the structure created due to divission of the surface into u = 5 parts and into v = 25 parts, the mass was equal to 1368.62 kg However, the distribution of internal forces at the supports for such a structure was uneven [38].

**Figure 2:**
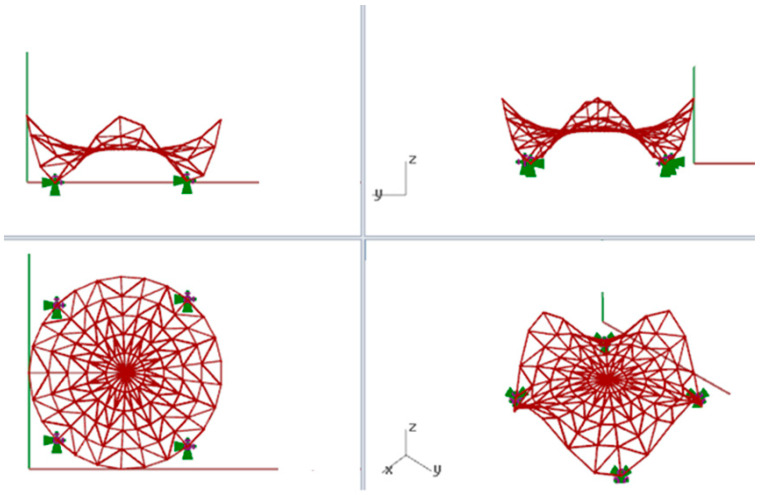
The views of the considered model of the curvilinear steel rod structure created on the basis of the Enneper surface.

Due to this fact, the subject of the analysis in the present article is a curvilinear steel rod structure based on the Enneper surface with four supports. Moreover, for further calculations, it has been assumed that the structure covers a round place whose radius is equal to 5.0 m, whereas the height of the structure is equal to 4.5 m, Figure 2. The structure was covered by glass panels, whereas as the structural material steel S235 was used. Rigid joints in the grid and pinned joints at supports have been applied.

**Figure 3:**
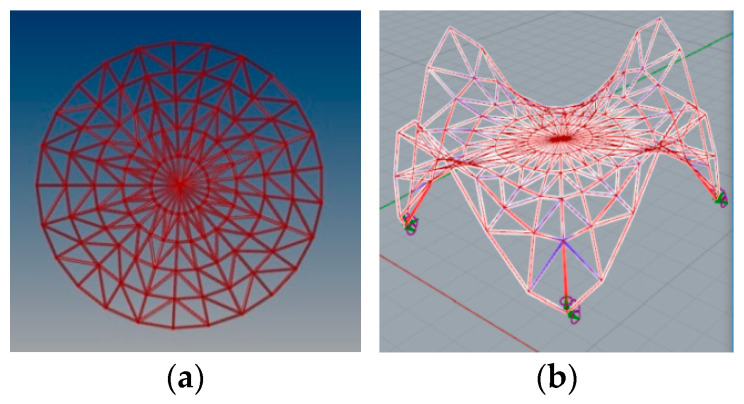
An initial model of the steel grid structure: (**a**) a rectangular projection; (**b**) distribution of stresses.

**Figure 4:**
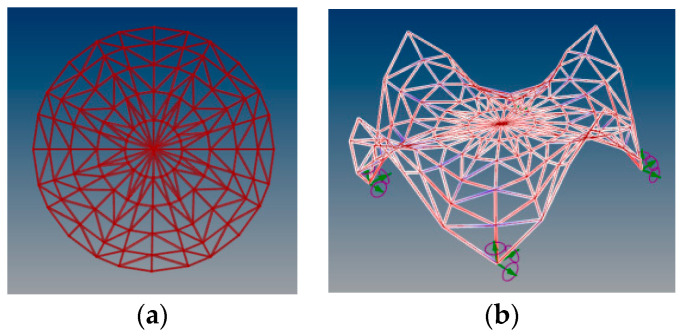
A modified model of the steel rod structure in order to reduce stress: (**a**) a rectangular projection; (**b**) distribution of stresses.

However, in order to create an efficient and a rational roof steel grid structure, further modifications have been applied thanks to the parametric description of the model. In order to unify the lengths of the structures’ rods, the base surface for creation of grids has been divided into an even number of segments along a boundary circle, that is, into v = 24 parts. Structural analysis of the structure under self load performed by Robot Structural Analysis Professional software has shown that the change of nodes’ positions cosed by symmetrical division influences the unification of stress distribution in rods at supports, as shown in Figure 3 and Figure 4.

**Figure 5:**
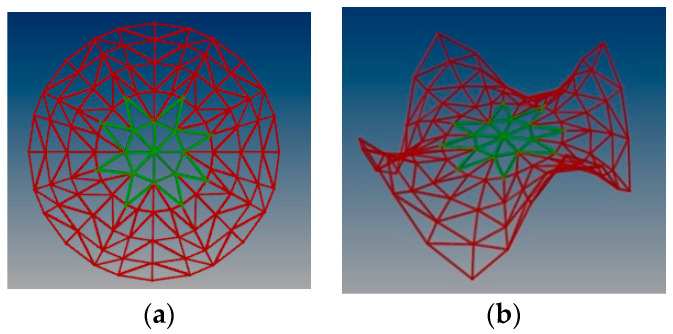
Modification of the model of the steel rod structure in order to minimize its mass: (**a**) the model’s horizontal projection; (**b**) the model’s perspective view.

In order to unify the size of panels, provide greater light access and reduce the mass of the structure, some of the rods were removed in the upper part of the structure, Figure 5.

**Figure 6:**
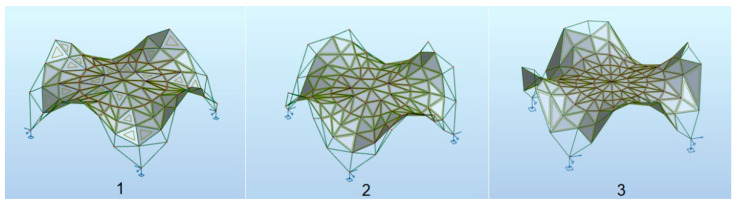
Various models of rod structures based on the Enneper surface.

The upper part of the structure has been raised to provide a 5% drop for easier drainage of rainwater. Moreover, a minimum number of panels has been introduced so as to cover the considered area. The highest external parts of the structure, which are mainly decorative, have been slightly changed in order to minimize the obstacles due to the snow load. Finally, various free solutions of the canopy roofs based on the Enneper surface have been proposed. The individual roofs differ in the number of elements and nodes as well as the number and the arrangement of the panels. They are called: structure 1, structure 2 and structure 3, as shown in Figure 6.

As with any structure, the main criterion for shaping the curvilinear steel rod structure is reliability. Due to this fact, the structural shaping proposed in the paper is based on European Standards included in Eurocodes [2,3,4,5]. According to the basic requirements specified there, a structure has to be shaped in such a way that meets conditions preventing failure as well as conditions that guarantee proper performance of it conceived for a pre-determined service life. The above means that Ultimate Limit States (ULS) and Serviceability Limit States (SLS) should be verified during the shaping process. Therefore, both ULS corresponding to states associated with failure of the structure and SLS corresponding to limit states of deformation have been verified during research. For that reason, each structure has been subjected to structural analysis using Autodesk Robot Structural Analysis Professional software and optimized due to its mass. As the material structural steel S235 was used, and glass panels with the same density and thickness as mentioned in Section 2. The models were treated as a combination of shells and beams.

The boundary conditions regarding snow and wind loads have been established for the structures located in Rzeszow, Poland. The structures were statically definite, and nonlinear analysis was applied.

In this analysis, two snow load cases have been considered: with an even snow load and with the possibility of snowdrift.

However, for each structure, the following input data have been adopted for analysis [4,42]:

- Characteristic value of snow load on the ground s_k_ = 1.2 kN/m^2^;
- Roof’s shape coefficient μ_1_ = 0.8 in case of even snow load;
- Roof’s shape coefficient μ_2_ = 2h/s_k_, in the case of snowdrift, where h is the height of the obstruction.

For structure 1 and structure 2, it has been established that μ_2_ = 2, and for structure 3, μ_2_ = 1.87, whereas various wind load cases have been generated automatically taking into account eight various wind directions and assuming a base wind velocity pressure equal to 0.3 kN/m^2^.

**Figure 7:**
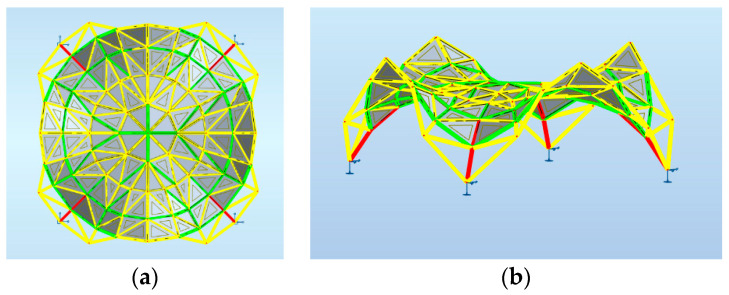
Structure 1 distribution of axial force Fx: (**a**) horizontal projection; (**b**) perspective view.

**Figure 8:**
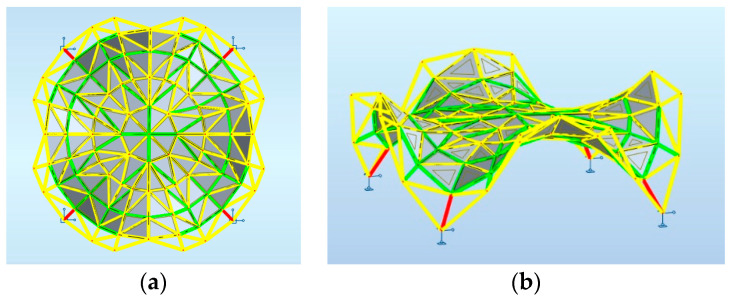
Structure 2 distribution of axial force Fx: (**a**) horizontal projection; (**b**) perspective view.

**Figure 9:**
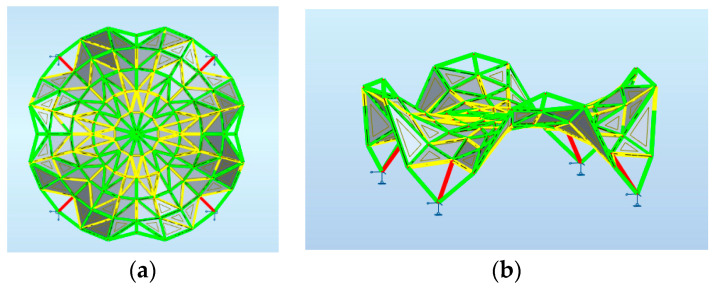
Structure 3 distribution of axial force Fx: (**a**) horizontal projection; (**b**) perspective view.

Maps on bars presenting distribution of the axial force Fx with the same scale applied for structures 1, 2, 3 are shown, respectively, in Figure 7, Figure 8 and Figure 9.

The rods of each structure were divided into two groups: support rods and the rest, according to the distribution of forces in the rods. Both the Ultimate Limit State and Serviceability Limit State were verified; however, the maximum utilization of structural members presented in Table 1, Table 2 and Table 3 is due to ULS. The results of the optimization of each structure at the mass acceptance as the optimization criterion are presented below in Table 1, Table 2 and Table 3.

However, the maximum values of structures’ deformations are as follows:

- Structure 1–4 mm,
- Structure 2–8 mm,
- Structure 3–4 mm.

On the other hand, maximum and minimum values of internal forces and moments for considered structures 1, 2, 3 are given, respectively, in Table 4, Table 5 and Table 6.

### 3.2. Shaping of Steel Rod Structures Based on Minimal Surfaces Defined by Two Pairs of Circles’ Arcs

#### 3.2.1. The Steel Rod Structure Based on Single Shell Surface

**Figure 10:**
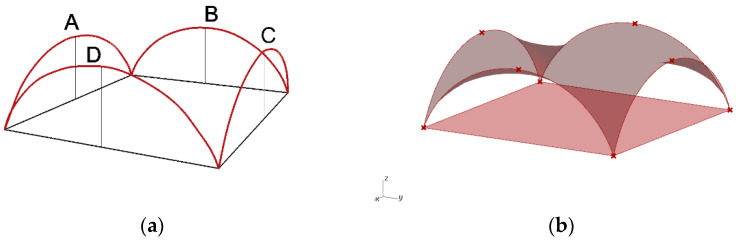
The minimal surface spanned on four identical arches of circles: (**a**) assumptions; (**b**) result.

The minimal surface stretched between four semicircle arches has been assumed as the basis for shaping the curvilinear steel rod structure being an open covering over the square place. Next, a Grasshopper algorithm for such a minimal surface creation with changeable parameters of the length of the square side and the heights of the arcs has been established. The arcs are to be contained in the vertical planes passing through the edges of the square, as shown in Figure 10a. For further calculations, it has been assumed that the length of the square’s side is equal to 24 m and the initial height of all arches is equal to 8 m. For these assumptions, the minimal surface has been stretched between the arches, as shown in Figure 10b.

The shape of this surface is not the most optimal shape for the roof covering because the roof in the middle part is almost flat, which may be unprofitable due to the snow load and rainwater drainage. Therefore, this initial surface has been modified. Namely, in order to improve the shape of the roof, the height of two opposite arches going through points A and C have been lowered, as shown in Figure 10a. For that reason, the defined script for surface generation has been used to run the simulation. Next, due to applied genetic surface optimization with minimal surface area as the optimization criterion and the height of the opposite arches as the optimization variable, the optimal minimal surface shape has been established.

**Figure 11:**
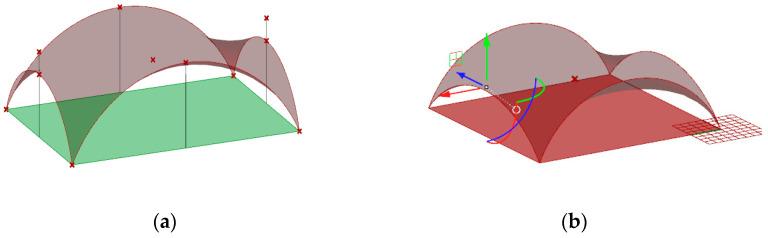
The minimal surface spanned on four arches: (**a**) assumptions; (**b**) reduction in the height of arches.

**Figure 12:**
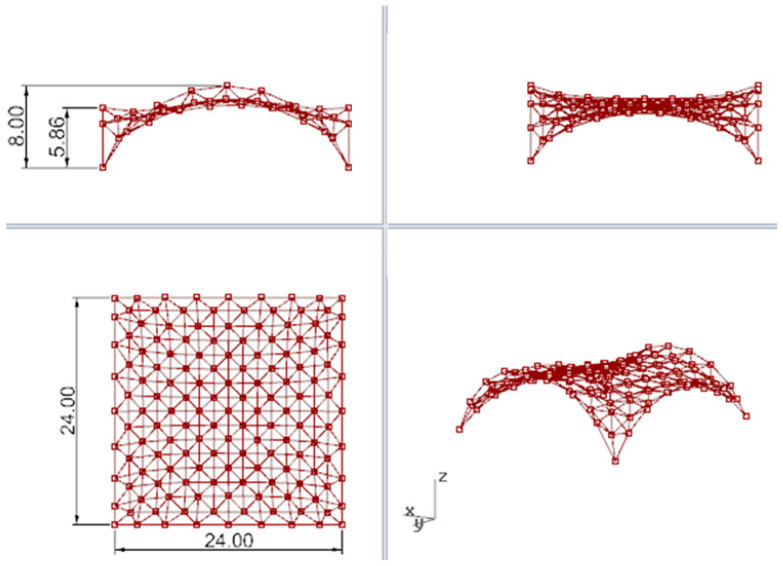
The views of the considered curvilinear steel rod structure.

Based on the optimization performed, the wanted height of these two arches has been set as 5.86 m. In this way, the new minimal surface is determined by two arches with a height of 8.0 m and two arches with a height of 5.86 m, as shown in Figure 11a. With this height of arches, the new minimal surface obtained has a surface area differing only 1 m^2^ from the original surface area, but its shape is much more favourable. However, with a greater reduction in the height of arches, the surface area increases significantly, and snow obstacles could form at higher arches, as shown in Figure 11b. Thus, the resulting surface is the minimal surface stretched on two arches of circles and two elliptical arcs. The optimal surface has been divided into eight equal parts along both directions, which guaranteed a symmetrical structure as well as reasonable rod lengths. Next, on the achieved grid, a 3D truss has been applied. The height of the truss was assumed as equal to 0.5 m. The truss has been located in such a way that the nodes of the upper part of it are contained in the base surface. The views of the achieved curvilinear rod structure are presented in Figure 12.

**Figure 13:**
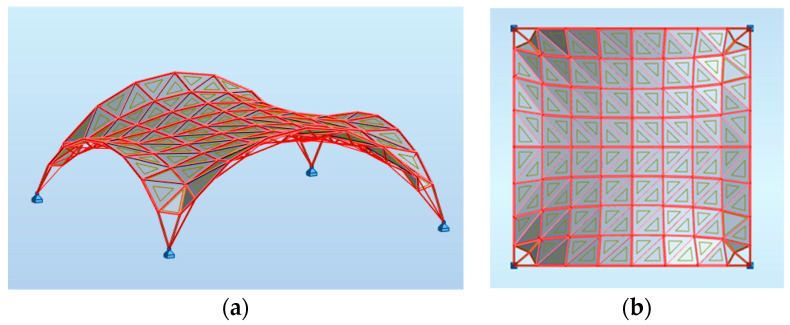
The way of placing the panels on the structure: (**a**) perspective view; (**b**) horizontal projection.

Further optimization of the structure has been carried out using Robot Structural Analysis Professional software. Similar to the structures based on the Enneper surface of the previous case studies, both ULS and SLS have been verified during the shaping process. For the analysis, it has been assumed that the kind of material is structural steel S235 and cross-sections for both the lattice and columns are circular hollows with wall thicknesses not less than 3.2 mm. In order to minimize the whole weight of the structure, polycarbonate plastic sheets have been used as cladding for roofing of density equal to 0.02 kg/m^3^ and panel thickness of 10 mm. The cladding is applied in such a way that the rods at the supports are exposed, as shown in Figure 13.

**Figure 14:**
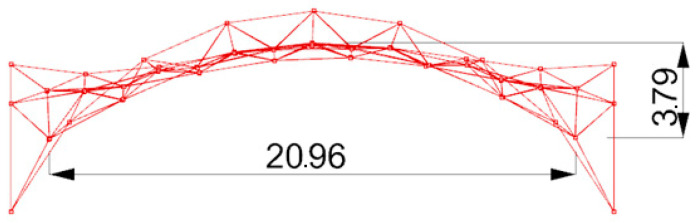
Dimensioning of the structure’s vertical view in order to establish the roof’s shape coefficient.

The boundary conditions regarding snow and wind loads have been established for the structures located in Rzeszow, Poland [4,42]. Two cases of snow loads are used for calculations: uniform and uneven, where the roof’s shape coefficients have been assumed as cylindrical roofs, according to the dimensions presented in Figure 14:

- Roof’s shape coefficient μ_1_= 0.8 in the case of even snow load,
- Roof’s shape coefficient μ_3_ = 0.2 + 10 h/b ≤ 2, in the case of uneven snow load,

where h = 3.79 m, b = 20.96 m, so μ_3_ = 2.0.

However, the wind load has been generated automatically assuming base wind velocity pressure equal to 0.3 kN/m^2^.

The structure has been optimized due to the worst case scenario resulting from the combination of loads. As the optimization criterion, the minimum mass of the structure was taken. Due to uneven distribution of stresses in rods, the rods of the structure have been divided into four groups for dimensioning: top truss rods, bottom truss rods, truss diagonal rods and rods at supports. The type of rod that has been used is round pipes. The final structure is composed of 145 nodes and 512 members.

**Figure 15:**
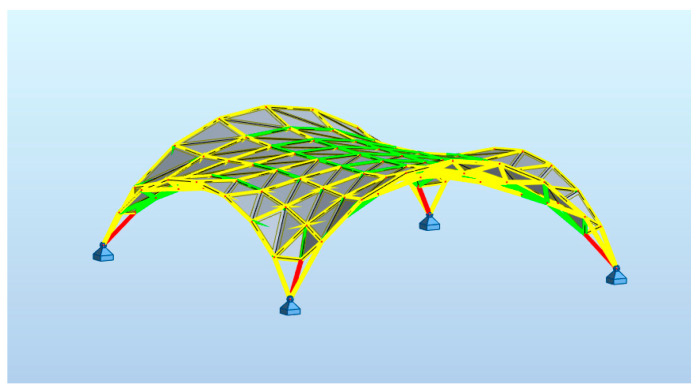
Maps on bars distribution of axial force F_X_.

Figure 15 shows that the middle bars at the supports are the most heavily loaded. The optimization criterion in the shaping of cross-sections was the mass of the structure. Table 7 shows the results of structural optimization, when all rods at supports have the same diameter. Attempts have been made in order to optimize the structure with the use of various cross-sections for support rods, but no significant weight reduction was achieved.

However, the maximum value of deformations was equal to 3 mm.

#### 3.2.2. The Steel Rod Structure Based on Multi-Shell Surface

**Figure 16:**
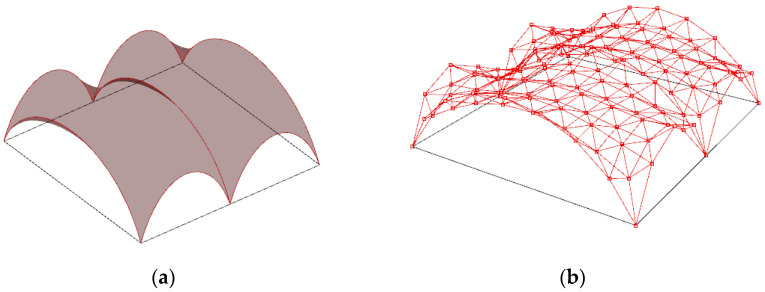
The two-layer grid structure: (**a**) a multi-shell-based surface; (**b**) a rod structure.

In the next stage, a curvilinear steel rod structure based on a double-shell surface has been applied as a covering structure over the given square. In this case, the base surface for the creation of the steel rod structure is the sum of two identical shells. Each shell is a minimal surface formed, such as the surface considered in Section 3.1 and it is presented in Figure 16. Due to this fact, the starting assumptions for creating the single shell are two arches, one of which is an arc of the circle and the other one the elliptical arc. The heights of the arches are identical to those of the surface considered earlier, that is, 8.0 and 5.86 m high. However, the span of the elliptical arc is two times smaller than in the previous case. Figure 16a,b shows, respectively, the multi-shell-based surface, as well as the curvilinear steel, rod structure shaped on its base, which is the subject of the present analysis.

**Figure 17:**
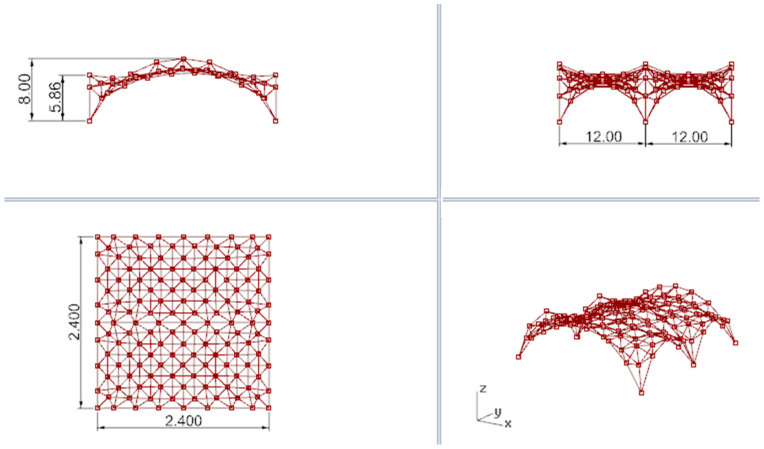
The views of the analyzed multi-shell curvilinear steel rod structure.

In the considered structure, a spatial truss of the same type as in the structure considered earlier in Section 3.1 has been sued. The height of the truss equals 0.5 m. The views of the analyzed curvilinear steel rod structure are presented in Figure 17.

**Figure 18:**
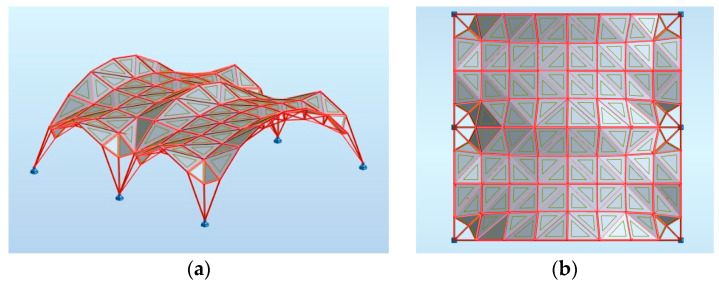
The way of placing the panels on the structure: (**a**) perspective view; (**b**) horizontal projection.

In order to compare the properties of the considered structure with the properties of the structure presented in Section 3.1, the same material was used for roofing, that is, polycarbonate plastic sheets, as well as round pipes for structural members of the same steel S235 material. The plastic sheets covering the roof have been arranged, as shown in Figure 18.

In the structural analysis performed by Robot Structural Analysis Professional software, two snow load cases have been considered, calculated for a cylindrical roof that has:

- An even load case with roof’s shape coefficient μ_1_ = 0.8;
- An uneven load case roof’s shape coefficient μ_3_ = 2.0.

**Figure 19:**
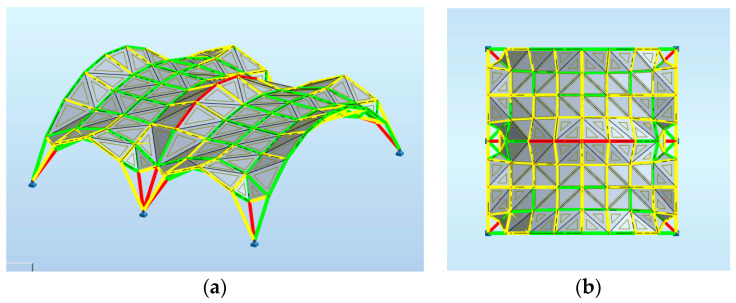
Maps on bars distribution of axial force *F_X_*: (**a**) axonometric view; (**b**) horizontal projection.

However, the opportunity to form snowdrifts due to protruding parts of the roof has also been taken into account. As a result of the analysis carried out, maps on bars have been obtained, showing the distribution of the force component *F*_X_, as shown in Figure 19.

The structure is composed of 520 rods and 154 nodes. The results of the structural optimization of the structure divided in several groups of rods are presented in Table 8. As the optimization criterion, the structure’s mass was taken.

Attempts to optimize the dimensions of the structure by creating a group of the most strenuous rods have not brought about a reduction in the mass of the structure. However, the maximum value of deformations of the structure equals 4 mm.

## 4. Discussion

Three proposals for steel-rod roof structures created on the basis of the Enneper surface covering the same round area have been presented. As the structural material, steel S235 was used. The structures have been created based partly on the results of the previous research on the steel structures of the Enneper forms presented in [38], where the efficiency of the steel rod structures is compared with three, four and five supports. The structure with four supports proved to be the most effective, and this kind of Enneper-like structure has been considered in the current research.

The shapes and topologies of the structures were to ensure fairly even distribution of stresses in the rods, which results in a reduction of the cross-sections of the rods and, consequently, lower weights of the structures. In order to reduce stresses at the supports, the division into an even number of parts along the perimeter of the covered place was used when creating surface meshes. The division into 5 parts along the radius and into 24 parts along the covered places’ perimeter for each structure was applied. This ensured a more even distribution of stresses in the supporting rods compared with the distribution of stresses in the supporting rods of the structures with the division into 25 parts along the perimeter.

Next, the form of each structure was adapted to the roofing shape and function. Three of the created structures differed in the number of rods, nodes and panels used. Their masses were optimized assuming dead and environmental loads. The most effective structure was selected in terms of weight, number of rods and the number of nodes. Comparing the results of the structural analysis performed for each structure, which are given in Table 1, Table 2 and Table 3, it can be concluded that structure 3 is the heaviest, whereas structure 1 is the lightest. Moreover, structure 1 is the most effective due to mass, the number of rods and the nodes applied. The analysis of the internal forces and moments acting on the structural members due to applied loads showed that all three steel rod structures based on the Enneper surface are effective because the stresses are mostly transferred by axial forces, as shown in Table 4, Table 5 and Table 6. The bending moments are small, which is very important for single-layer structures with rigid joints. Moreover, the research showed that small modifications of the initial structure by shifting its vertices beyond the base surface in order to adapt the structure to the roof function did not significantly affect its effectiveness. This is promising for shaping other forms of structures based on the Enneper surface.

The other generated and analyzed structures were both single shell and double shell steel rod structures based on minimal surfaces stretched on four arcs. In this case, the base surface area was minimized and adapted to the roof function before its discretization. Then, a 3D truss was applied with an even number of divisions on both single shell and double shell surfaces covering the same area. A comparative analysis of single and double-shell structures was performed. According to Table 7 and Table 8, the mass of the single shell structure is almost twice as low as the mass of a structure based on a double-shell surface. This difference may be caused by a smoother surface of a single shell structure and, thus, the lack of the possibility of depositing snow on the roof.

Due to the fact that the proposed steel rod structures both based on the Enneper surface and on the free form surface are new and original, it is difficult to find similar engineering solutions to compare them within the literature. The structural shaping proposed in the paper and treated as a successive optimization of the structure’s shape in order to obtain more efficient structures meets the conditions of both Ultimate Limit States ULS and SLS. The analysis has been performed on optimized virtual models, which helped to choose the best solution due to structure shapes and topologies. Due to the fact that the same principles and criteria were applied to each structure, the obtained optimization results can be considered reliable. In order to verify the obtained results, the experiments with physical models of the best solutions of both kinds of structures are planned in the future as well calculations of joints, which are very important for the structure’s strength [43].

**Figure 20:**
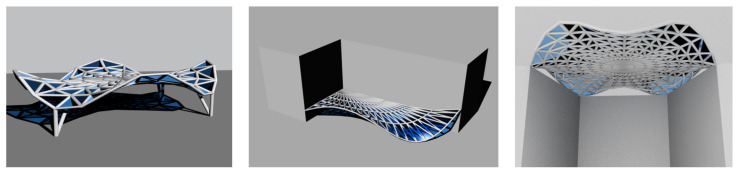
Various models of steel rod structures based on the Enneper surface.

However, the research on shaping structures on the basis of minimal surfaces has shown that the obtained structures, as a result of both geometric and structural optimization, may constitute proposals of effective steel rod structures and find practical applications. Moreover, algorithmic-aided shaping of structures supports the shaping process as after changing the parameters that define the considered structures, other solutions of steel rod structures that are interesting in terms of form and mechanical properties can be obtained, which can also find practical use. Such structures created on the basis of the Enneper surface are presented in Figure 20.

The obtained structural models as the result of the conducted research are economical not only due to the low weight of the structural material used but also due to the use of minimal area for claddings. Due to this fact, the research is worth continuing and extending in order to shape more complex structures as well as structures composed of several units.

## 5. Conclusions

An algorithmic-aided method of shaping curvilinear steel rod structures based on minimal surfaces has been proposed in order to find original and efficient structures. The application of this method has been shown on the examples of shaping structures based on the Enneper surface as well as based on free form surfaces determined by four arcs. The method of shaping structures, treated as the successive optimization of their forms in order to obtain the most effective structures, combines the application of the tools allowing generation of the complex geometric models as well as the tools that enable proper finite element analysis. Several steel rod structures of the minimal surface form have been generated, analyzed and compared. The research has shown that the considered structures are effective in terms of load transfer. They have been optimized using mass as the optimization criterion, which significantly determines the cost of the structure. However, the author is aware that in the case of curvilinear steel rod structures, it is not sufficient to adopt their masses as the optimization criterion. In the case of these structures, it is also important to standardize the length of the rods as well as to calculate and design joints. Due to the fact that shaping of structures is an initial design phase, these aspects have been omitted in the research. In addition to creating and analyzing original curvilinear steel rod structures, the research has shown how to search for such structures at the first stage of the design by using modern digital tools. The paper presents theoretical research that forms the basis for further experimental research.

## Figures and Tables

**Table 1:** Dimensioning of the considered roof structure 1 due to structural optimization.

Kind of the Structural Member	Circular Hollow Section (mm/mm)	Maximum Utilization Due to ULS (%)
Support rods	60.3/3.6	97
Other rods	54.0/3.2	97

Total mass 1219.57 kg, 94 nodes, 252 rods.

**Table 2:** Dimensioning of the considered roof structure 2 due to structural optimization.

Kind of the Structural Member	Circular Hollow Section (mm/mm)	Maximum Utilization Due to ULS (%)
Support rods	60.3/3.6	97
Other rods	60.3/3.2	96

Total mass 1519.61 kg, 102 nodes, 260 rods.

**Table 3:** Dimensioning of the considered roof structure 3 due to structural optimization.

Kind of the Structural Member	Circular Hollow Section (mm)	Maximum Utilization Due to ULS (%)
Support rods	70.0/3.2	88
Other rods	70.0/3.2	88

Total mass 1826.73 kg, 109 nodes, 300 rods.

**Table 4:** Maximum and minimum values of internal forces and moments of the structure 1.

	Fx(kN)	Fy(kN)	Fz(kN)	Mx(kNm)	My(kNm)	Mz(kNm)
Max	74.6	0.66	1.89	0.12	0.72	0.40
Min	−23.22	−0.62	−2.07	−0.14	−0.93	−0.39

**Table 5:** Maximum and minimum values of internal forces and moments of the structure 2.

	Fx(kN)	Fy(kN)	Fz(kN)	Mx(kNm)	My(kNm)	Mz(kNm)
Max	82.35	0.77	2.00	0.12	0.77	0.45
Min	−32.00	−0.74	−2.36	−0.12	−1.04	−0.44

**Table 6:** Maximum and minimum values of internal forces and moments of the structure 3.

	Fx(kN)	Fy(kN)	Fz(kN)	Mx(kNm)	My(kNm)	Mz(kNm)
Max	78.70	1.3	2.31	0.32	1.23	0.89
Min	−30.92	−1.3	−2.3	−0.32	−1.7	−0.88

**Table 7:** Dimensioning of the considered roof structure due to structural optimization.

Kind of the Structural Member	Circular Hollow Section (mm/mm)	Maximum Utilization Due to ULS (%)
Truss top rods	127.0/4.0	91
Truss bottom rods Truss diagonals Columns	193.7/5.0 101.6/3.6 273.0/5.0	96 94 88

Total mass 19,676.23 kg.

**Table 8:** Dimensions of the considered roof structure due to structural optimization.

Kind of the Structural Member	Circular Hollow Section (mm/mm)	Maximum Utilization Due to ULS (%)
Truss top rods	273/5.0	87
Truss bottom rods Truss diagonals Columns	219.1/5.0 117.8/5.0 323.9/5.0	98 98 98

Total mass 38,273.33 kg.

## Data Availability

Data are contained within the article.
